# Isolation and characterization of two plasmids in a clinical *Acinetobacter nosocomialis* strain

**DOI:** 10.1186/1756-0500-7-732

**Published:** 2014-10-17

**Authors:** Bianca Gifford, Joseph Tucci, Simon J McIlroy, Steve Petrovski

**Affiliations:** La Trobe Institute for Molecular Sciences, La Trobe University, Bendigo, Australia; Molecular Pathology, Peter MacCallum Cancer Centre, St Andrews Place, East Melbourne, 3002 Australia

**Keywords:** Cryptic plasmid, *Acinetobacter baumannii*, pRAY, pAB49, *Acinetobacter nosocomialis*

## Abstract

**Background:**

*Acinetobacter* species are recognised as important nosocomial pathogens that have become a major cause of invasive opportunistic infections in hospitalised patients. Their clinical significance is largely due to the rapid development of antimicrobial resistance among strains. The development of antibiotic resistance among bacterial strains occurs frequently by the acquisition of resistance genes by gene transfer systems such as bacterial plasmids.

**Method:**

Multi-antibiotic resistant *Acinetobacter nosocomialis* strain 178 was isolated from a hospital in Melbourne, Australia. This strain was screened for the presence of plasmids. The two plasmids isolated were sequenced and annotated.

**Results:**

Two plasmids isolated from a single clinical *Acinetobacter nosocomialis* strain were sequenced. One plasmid, designated pRAY*-v3, appears to have evolved *via* the same lineage as the pRAY plasmid isolated from an *Acinetobacter baumannii* in South Africa. The other plasmid, designated pAB49-v1, appears to be an evolutionary descendent from a cryptic plasmid isolated from an *A. baumannii* almost 20 years ago. Both of the plasmid sequences here share a high level of sequence similarity with their ancestors, however differences are noted.

**Conclusion:**

The isolation of these plasmid-lineages across different decades and continents suggests their global dissemination.

## Background

The *Acinetobacter calcoaceticus-baumannii* (ACB) complex [1] consists of four closely related genospecies, namely *A. calcoaceticus* (genomic sp. 1), *A. baumannii* (genomic sp. 2) [2], *A. nosocomialis* (former genomic sp. TU13) and *A. pittii* (former genomic sp. 3) [3], along with two additional unnamed genomic species [4]. These species, particularly *A. baumannii, A. nosocomialis* and *A. pittii,* are recognised as important nosocomial pathogens that have become a major cause of invasive opportunistic infections among critically ill hospitalised patients [5, 6]. Their clinical significance is largely implicated by the rapid development of multi-antimicrobial resistance among these species, making hospital-acquired infection difficult to treat [5, 6]. The development of antibiotic resistance among bacterial strains occurs frequently by the acquisition of resistance genes by gene transfer systems, such as bacterial plasmids, transposable elements and integrons, where plasmids serve as the vehicles for resistance gene capture and their subsequent dissemination throughout the microbial population [7]. Bacterial plasmids are commonly observed among members of the *Acinetobacter* genus where they are suggested to play an important role in the adaptive evolution of these organisms [7–9]. Considering this suggested role for plasmids, coupled with their implication in serious antibiotic resistance issues in hospitals, makes further study into the detail of their functions of paramount importance.

We here report the isolation and characterization of two plasmids isolated from a single clinical strain of *A. nosocomialis* obtained from a Melbourne hospital.

## Methods

The bacterial strain *Acinetobacter* sp. strain 178 was provided to us from a local Melbourne (Australia) hospital and is the subject of this study. The source of the strain is unknown and de-identified. The *Escherichia coli* K-12 derivative used for transformation of cloned DNA fragments was DH5α (*Δlac169 endA1 recA1* Res^-^ Mod^+^ Nal^r^) [10]. The plasmid used for cloning was pBluescript KS+. Bacteria were grown on Luria-Bertani (LB) medium and incubated at 30 °C for *Acinetobacter* strains and 37 °C for *E. coli*. To select *E. coli* strains with conferred resistance (or *A. nosocomialis* strains, as indicated) antimicrobials were added to LB at the following final concentrations (*μ*g mL^-1^): ampicillin (Ap) 50 and 100; chloramphenicol 32; tetracycline 80; kanamycin 12.5; rifampicin 2; gentamycin 10; streptomycin 10.

To determine the identity of the clinical isolate str. 178, the 16S rRNA gene and 16S-23S Internal Transcribed Spacer (ITS) region were PCR amplified using the primers 6R (GGGTTYCCCCRTTCRGAAAT) [11] and 27 F (GAG TTT GAT CMT GGC TCA G) [12]. Sequencing was performed by Australian Genome Research Facility (AGRF) (Brisbane, Australia) using the primers 530 F (GTG CCA GCM GCC GCG G), 519R (GWA TTA CCG CGG CKG CTG), 1114 F (GCA ACG AGC GCA ACC C), 907R(CCG TCA ATT CMT TTR AGT TT) [12], 1512 F (GTCGTAACAAGGTAGCCGTA) and 6R (GGGTTYCCCCRTTCRGAAAT) [11]. Phylogenetic placement was assessed with the alignment and construction of a phylogenetic tree of related sequences [13]. The ITS region was also sequenced to complement 16S rRNA gene analysis, as the 16S rRNA gene is reportedly unable to provide phylogenetic resolution between species of the ACB complex [14].

Plasmids were isolated, digested with restriction endonucleases *Hin*dIII and the fragments were cloned into pBluescript KS+. The fragments were sequenced and assembled, and primer walking was used to close any remaining gaps. The fragments were sequenced using pUC/M13 forward and reverse primers [15]. Cloning, plasmid DNA isolation and other manipulations were performed as described previously [16]. DNA sequence analysis was performed using the Geneious Pro 4.0.4 and the BlastN algorithm at National Centre Biotechnology Information (NCBI).

PCR amplification employed novel primers, BG1 (CGATTTATGTCAGTTCGCGG) and BG2 (CTCTGAGTTGGCTGACAATG), to amplify a region within pAB49 and its relatives using the conditions described in Petrovski & Stanisich, [16].

The nucleotide sequence of the 16S rRNA gene and ITS region has been deposited in GenBank under accession number KC257034. The plasmid sequences for pRAY*-v3 and pAB49-v1 have been deposited in GenBank under the accession numbers KC417494 and KC417495 respectively.

## Results and discussion

*A. nosocomialis* strain 178 was selected from 41 clinical *Acinetobacter* strains collected from a local Melbourne (Australia) hospital. This strain was of particular interest because of its relatively broad antibiotic resistance profile, growing in the presence of gentamicin, streptomycin, chloramphenicol and kanamycin. Gel electrophoresis of plasmid DNA isolated from strain 178 indicated the presence of two small plasmids ~6 kb and ~2.2 kb in size. The plasmids were sequenced revealing they were 6,078 bp and 2,200 bp in size, correlating well with the estimated sizes. Both plasmids had a GC mol% content of 39%, which is similar to the genome of their host species [17], and are here named pRAY*-v3 and pAB49-v1.

**Figure 1:**
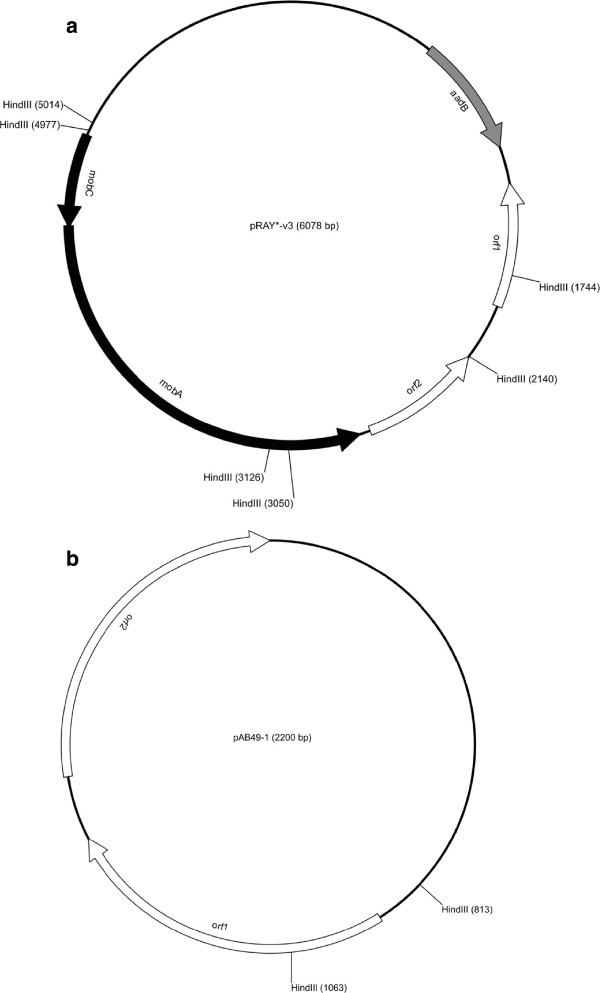
**Genetic organization of isolated plasmids. (a)** Genetic map of pRAY*-v3 **(b)** Genetic map of pAB49-v1. Arrows represent putative open reading frames. HindIII sites are indicated on plasmid maps.

The compiled sequence of the 6,078 bp plasmid is almost identical to the sequence of the plasmid pRAY from *Acinetobacter sp.* SUN, which was isolated in the 1990s in South Africa [18]. More recently plasmids similar to pRAY with small variations have been isolated from *A. baumannii* strains in New South Wales (Australia) and named pRAY*, pRAY*-v1 and pRAY*-v2 [19, 20]. We therefore here name this plasmid pRAY*-v3. pRAY*-v3 is most closely related to pRAY*-v1 with only a single nucleotide substitution at position 1656 from a C > G. The other two Australian isolates pRAY* and pRAY*-v2 share 99% identity with pRAY*-v3. Comparison of pRAY*-v3 with both pRAY* and pRAY*-v2 reveals that an additional 55 to 60 bp substitution occurs within the *mobA* gene (Figure 1a). pRAY*-v2 also contains an insertion of a putative IS-element [20].

Our annotation of the pRAY*-v3 plasmid reveals five putative ORFs designated as *aadB, orf1, orf2, mobA* and *mobC* (Figure 1a). The *aadB* gene encodes an aminoglycoside resistance protein, enabling the host to have a competitive advantage at survival in the presence of such antimicrobials. However, this gene is generally located within the *attI* site of integrons and appears to be a gene cassette. Despite this finding, no integrase gene is present in pRAY*-v3 so the integration of this gene cassette probably occurred when an integrase was provided *in trans* from another DNA molecule [21]. The DNA sequence upstream of the *aadB* gene must also encode a promoter region, as gene cassettes traditionally solely rely on the Pc promoter, located within an integrase, for their expression [21]. Two putative genes of unknown function are present downstream of the *aadB* gene, here named *orf1* and *orf2*. Following these two genes are two additional genes, *mobA* and *mobC,* thought to encode putative mobilisation proteins. The presence of such proteins suggests that the pRAY plasmids could be mobilisable and the presence of an IS-element in pRAY*-v2 suggests that these pRAY plasmids could potentially act as vehicles for the dissemination of transposable elements in *Acinetobacter* species, particularly the ACB complex.

Comparisons of pRAY*-v3 with its close relatives reveals most mutations have occurred within the *mobA* gene, suggesting that this gene is either not essential for plasmid replication or maintenance or that these mutations have no affect on the function of the encoded protein. This plasmid and its derivatives have been reportedly detected in various locations around the world that include South Africa, Australia, Europe and the USA [20] suggesting stable maintenance within *Acinetobacter* strains. This is the first reported isolation of a pRAY lineage plasmid from *A. nosocomialis,* having previously only been detected in *A. baumannii* strains, suggesting that the pRAY-lineage plasmids are at least more widely distributed among members of the ACB complex.

The DNA sequence of the smaller plasmid (2,200 bp), isolated from *A. nosocomialis* str. 178*,* was compared to other DNA sequences in GenBank and appears to be 78% similar to the first 1850 bp of the DNA sequence of plasmid pAB49 (Acc No L77992), deposited in the database in 1996 but is as yet unpublished. The remaining 350 bp of the sequence are novel. We therefore here named this plasmid pAB49-v1.

The identification of pAB49-v1 15 years after the sequence submission of pAB49, without any apparent selection pressure, is interesting and suggests that perhaps these pAB49 type plasmids are stably maintained in some *Acinetobacter* strains. The plasmid described here encodes two putative genes presumably involved in plasmid replication (Figure 1b).

To determine the distribution of the plasmid among related clinical ACB complex strains, we designed primers that would amplify a 1 kb region of its sequence and conducted PCR on 41 *Acinetobacter* isolates from a local Melbourne hospital. Only the 178 strain gave a PCR product, indicating the presence of the pAB49-v1.

We cloned the entire pAB49-v1 into pBluescript KS + (see materials and methods). This newly created plasmid contained the origin of vegetation of the *E. coli* plasmid pBluescript KS + and that of the *A. nosocomialis* plasmid pAB49-v1. Furthermore the plasmid now had a selectable marker, namely ampicillin. To determine its host range we attempted to transform this plasmid into ampicillin sensitive (at 50 μg/ml) *Acinetobacter baumannii*. The plasmid was unable to replicate in the type strain suggesting a narrow host range potentially restricted to *A. nosocomialis*. Despite these findings, the detection of the plasmid in two separate continents 15 years apart indicates that this small plasmid may have disseminated between *Acinetobacter* strains throughout the world.

## Conclusion

In conclusion we isolated two plasmids from a clinical *A. nosocomialis* strain from Melbourne Australia. Ancestors of the two plasmids have been previously isolated and sequenced from different continents in *Acinetobacter* strains. Plasmid pRAY has a selective advantage to its host, providing a mechanism for resistance to aminoglycosides, whereas pAB49 does not. The fact that such plasmids can be isolated many years later, in geographically distinct locations, suggest they are strongly maintained within their hosts, despite the smaller plasmid having no evident selective advantage. The work presented here suggests that these two plasmids may be widely disseminated among members of the ACB complex throughout the world and thus could play an important role in their ecology.
